# Neutron activation of gadolinium for ion therapy: a Monte Carlo study of charged particle beams

**DOI:** 10.1038/s41598-020-70429-9

**Published:** 2020-08-07

**Authors:** Kurt W. Van Delinder, Rao Khan, James L. Gräfe

**Affiliations:** 1 Department of Physics, Faculty of Science, Ryerson University, 350 Victoria St., Toronto, ON M5B 2K3 Canada; 2 Medical Physics Division, Department of Radiation Oncology, Washington University School of Medicine, 660 S Euclid Ave, St Louis, MO 63110 USA

**Keywords:** Cancer, Particle physics, Cancer imaging

## Abstract

This study investigates the photon production from thermal neutron capture in a gadolinium (Gd) infused tumor as a result of secondary neutrons from particle therapy. Gadolinium contrast agents used in MRI are distributed within the tumor volume and can act as neutron capture agents. As a result of particle therapy, secondary neutrons are produced and absorbed by Gd in the tumor providing potential enhanced localized dose in addition to a signature photon spectrum that can be used to produce an image of the Gd enriched tumor. To investigate this imaging application, Monte Carlo (MC) simulations were performed for 10 different particles using a 5–10 cm spread out-Bragg peak (SOBP) centered on an 8 cm^3^, 3 mg/g Gd infused tumor. For a proton beam, 1.9 × 10^6^ neutron captures per RBE weighted Gray Equivalent dose (GyE) occurred within the Gd tumor region. Antiprotons ($\bar{P}$), negative pions (− π), and helium (He) ion beams resulted in 10, 17 and 1.3 times larger Gd neutron captures per GyE than protons, respectively. Therefore, the characteristic photon based spectroscopic imaging and secondary Gd dose enhancement could be viable and likely beneficial for these three particles.

## Introduction

The objective of depositing a therapeutic radiation dose to the tumor while minimizing damage to nearby healthy structures requires imaging modalities for accurate target localization during radiation treatment. Modern external beam radiation therapy uses collimated high energy photon beams in the form of either intensity modulated radiation therapy (IMRT) or volumetric modulated arc therapy (VMAT)^1^ to deliver radiation doses. Contrary to megavoltage photon beams, ion beams can provide an even higher dose to the tumor region while further reducing dose to healthy tissues beyond the tumor. Based on this premise, there has been an exponential increase in demand for systems employing the use of protons within clinical care in the last two decades. Heavy particles have a higher relative biological effectiveness (RBE) than protons, which may provide a better therapeutic ratio and therefore a clinical advantage. Globally, 12 centers are currently using carbon ion beams and many facilities are investing large amounts of funding to investigate the use of other particles for therapeutic purposes^2,3^.

In particle therapy, high energy particles interact with beam shaping components located within the treatment units, producing secondary neutrons through nuclear interactions. The initial neutron energies depend on the initial particle energies, density and composition of the interacting medium. The amount of materials in the path of the particle beam depends on the design of the treatment unit and the delivery method used. Passively scattered techniques may require several beam shaping components to be added, which may include a scatterer, modulator, collimator and range modulator. Magnetically scanned techniques use magnets to shape the pencil beams and therefore do not require added materials in-front of the beam path^4^. As a result, techniques that use magnets for beam placement, produce a smaller distribution of secondary neutrons. Secondary neutrons may also be produced from the interaction of the charged particle beam and the tissue of the patient that is irradiated. The two main nuclear processes responsible for the production of neutrons are: intranuclear cascade and nuclear evaporation^5^. When a high energy charged particle (> 50 MeV) with an impact parameter less than the atomic radius interacts with the target nucleus, neutrons in addition to other particles and light fragments can be driven out with high energy in the forward direction. This process is referred to as the intranuclear cascade. As a result of the missing nucleons, the excited nucleus decays from its excited state by the emission of low energy nucleons (neutrons) isotropically. As the neutrons traverse within the tissue of the patient, the two main types of interactions are neutron scattering and neutron absorption. The inelastic scattering process occurs when a high energy neutron (> 1 MeV) interacts with a target nucleus resulting in a change of system energy. Inelastic scattering results in an excited target nucleus which will destabilize by emitting gamma-rays. Elastic scattering is the primary mechanism responsible for higher energy neutrons to thermalize down to very low energies. The neutron absorption process occurs when a neutron is absorbed by a target nucleus. The nucleus will excite to a higher energy state and the de-excitation process may occur by several different mechanisms^5^.

Since the radiation quality factor of neutrons can reach as high as 20^6^, an increased healthy tissue damage from this scattered neutron distribution becomes more significant. Recently, a procedure has been proposed to inject a therapeutically beneficial neutron-capturing agent into the tumor. While the agent captures thermal neutrons, it may result in the production of photons for imaging and could enhance the local dose to the tumor, therefore, turning a negative byproduct into a positive therapeutic benefit^7,8^. The concept of imaging the unique signature photon spectrum produced from a material as a result of neutron activation is referred to as ‘neutron activated imaging’. The specific application of measuring the gadolinium neutron capture (GdNC) spectrum produced from proton therapy was previously termed Proton Neutron Gamma-X Detection (PNGXD)^8^. In principle, this approach can be extended to include all heavy charged particles, therefore, a more general term, Particle Neutron Gamma-X Detection PNGXD is being proposed here.

The prospective applications of PNGXD includes the production of a dynamic tumor image to be fused with an anatomic image modality such as CT or MRI and used as a method to track tumor position. The localization of the tumor volume could also be used as a method to determine treatment response and allow the incorporation of additional treatment customizations such as, adaptive planning techniques^9^. In order to produce a PNGXD nuclear medicine image, the addition of a spectroscopic detection system would be included with treatment delivery. The main GdNC spectral signature ranges in energy from 43 to 181.9 keV and is therefore ideal for most spectroscopic detection systems. The application of novel dynamic-SPECT (D-SPECT) systems may allow a smaller treatment room presence with little reduction in imaging quality, however, further experimental investigation is needed. Experimental measurements performed on a 5 × 5 mm^2^ cadmium telluride (CdTe) detector have documented signal-to-noise ratios as high as 15^9^. As proton range verification techniques such as positron emission tomography (PET) or prompt γ-ray emission also require the incorporation of a detection system, it may be possible to perform a PNGXD imaging procedure in-synchrony to a range verification technique^10,11^.

In this application, gadolinium would be injected into the patient prior to treatment. Gadolinium is comprised of seven naturally abundant isotopes, which have a total thermal neutron cross section of 48,800 barns (b). ^157^Gd (15.7% abundance) and ^155^Gd (14.8% abundance) have thermal neutron capture cross sections of 254,000 b and 60,900 b, respectively^12^. As a result of ^157^Gd neutron capture reaction, 7.94 MeV (Q-value) of energy is released with 99% of the Q-value energy given to prompt gamma-ray radiation^12^. The main spectral photon signature from this capture reaction produces γ-rays of 79.5 and 181.9 keV and characteristic X-rays of 43 keV (K_α_) and 49 keV (K_β_). Neutron capture with ^155^Gd also contributes to the characteristic X-ray production. The neutron capture cross sections and main spectral photon emission probabilities of interest to this study are listed within Table 1. The absorption of a neutron with a ^157^Gd nucleus produces internal conversion (IC) electrons with a total yield of 0.69 and average energy of 66.5 keV^13^. The discrete spectrum of IC electrons range in energy from 29 to 246 keV, with the most intense discrete emission occurring at 71 keV^14^.

**Table 1:** Gd isotopic abundance, neutron capture cross sections and photon emission probabilities per neutron capture, for the photon emissions of interest in this study.

Isotope	Percent abundance (%)	Thermal neutron capture cross section (b)		Photon emission energy (keV)	Photon emission probabilities (%)
${}^{157}Gd$	15.65	254,000 ± 800^a^	γ-ray	181.9	18.33 ± 1.69^b^
			γ-ray	79.5	9.75 ± 0.69^b^
			${K}_{\alpha }$	43	18.2 ± 1.0^c^
			${K}_{\beta }$	49	4.5 ± 0.3^c^
${}^{155}Gd$	14.80	60,900 ± 500^a^	${K}_{\alpha }$	43	29.1 ± 3.8^c^
			${K}_{\beta }$	49	7.1 ± 0.9^c^

^a^From IAEA^26^.

^b^From Kibédi^27^.

^c^From Gräfe et al.^28^.

The relaxation of ^158^Gd* compound nucleus also yields 5 Auger electrons with an average of 0.85 keV energy per capture and 0.84 X-rays with an average energy of 12.77 keV per capture^13,15,16^. The IC and Auger electrons contribute to a high linear energy transfer (LET) dose that is deposited locally within tissue. In addition to X-rays, as a result of the nuclear de-excitation, a high energy spectrum of γ-rays are emitted with a yield of 1.83 per neutron capture and an average energy of 1,330 keV^13^.

Several studies have demonstrated Gd as a dose enhancing neutron capture agent. The tumor uptake of Gd and relative biological effectiveness (RBE) of the neutron capture produced Auger and internal conversion electrons have been previously investigated. Cellular Gd uptake concentrations as high as 3,000 parts-per-million (PPM) without reaching any signs of cytotoxicity have been developed^17,18^. Cancer cells commonly possess an elevated mitochondrial membrane potential as compared to healthy cells, and therefore the synthesis of Gd^III^-triarylphosphonium salts have been developed. Within a feasibilitiy study, the novel Gd^III^ complexes were found to be tumor cell selective, mitochondrially-targeting and had a very high tumour-cell uptake^17,18^.

The most promising Gd compound that has been clinically studied, is the macrocyclic texaphyrin derivative known as Motexafin-Gd (MGd) which is currently undergoing a Phase III clinical trial. This is a strong candidate for GdNC for the application of whole brain radiation therapy^19^. Gd has a prolonged tumor retention of up to 2 months, however, the absorbed concentration was found to peak within a 6 to 12 h window^20^. Gd possesses a 70:1 (varying from 37 and 133) tumor-to-healthy tissue uptake ratio with a 90% uptake in glioblastoma cell nuclei^19–21^. The RBE of the neutron capture produced electrons have been found to vary from one study to another. An RBE of 12.6, 6 and 1.5 were quantified using a Monte Carlo (MC) toolkit along the inside, surface and outside of a 3 nm radius cylinder mimicking a DNA molecule^22^. By method of quantifying radiation weighting factors and relative biological effectiveness, RBE values of 5 and 20 were recommended for spherical cells and DNA, respectively^23^. An RBE of 10 was recommended by Humm et al. from a calculated review of Auger electron dosimetry for an emitter bound to the DNA of a nucleus^24^. These studies quantifying the RBE values, could take advantage of the recent advances made in MC simulation of biological nanostructures and microstructures^25^.

In a previous experimental study, the main photon spectrum for Gd was characterized for a passive scattering proton therapy beam^9^. In another study, gadolinium dose enhancement resulting from protons and carbon ion therapy was investigated via a MC simulation^7^. Since a greater number of neutrons are produced per particle from heavier ions, the quantification of both GdNC photon production and enhanced dose scaled per Gy is of great interest for particles with a larger charge and mass than protons. In addition, there are several other unique particles that have been explored for use in radiation therapy that may be of strong interest for the application of gadolinium neutron capture therapy.

Antiprotons ($\stackrel{-}{P}$) and negative pions (− π), exhibit unique interaction mechanisms that can result in an increased tumor dose however at the expense of large secondary neutron production. Antiprotons are similar to protons in terms of physical beam characteristics such as energy-range, stopping power ratios and RBE^29^ along the entrance plateau region. However, within the Bragg peak, antiprotons undergo antiproton-nucleon annihilation releasing approximately 2 GeV of energy. This annihilation leads to a doubling of physical dose in addition to an increased biological dose^29^. Negative pions have a mass of approximately 1/7th that of a proton, but still exhibit characteristics similar to other charged particles including the capability to produce a spread-out Bragg Peak (SOBP).

When a negative pion slows down within tissue, it may be captured by any of its elemental constituents producing a star formation of heavily ionizing fragments capable of depositing a large localized radiation dose^30^. When a negative pion slows down and is specifically captured by hydrogen, a unique feature occurs in which the particle is placed within the electronic orbit, similar to an electron. When this atom comes close to a heavier atom, the pion is transferred to the heavier element due to a lower binding energy. The pion becomes absorbed within the nucleus of the heavy element releasing a total of 140 MeV of kinetic energy, a phenomenon known as a “star formation” due to its star-like appearance in emulsion and cloud chambers^30,31^. Of this kinetic energy, ~ 70 MeV is carried by neutrons, 30 MeV by other charged particles such as protons, alpha particles and heavy particles and remaining 40 MeV to overcome the nuclear binding energy^30,31^.

To further study the role of GdNC and spectroscopic imaging, MC simulations were performed to determine which particle therapy would benefit the most from dose enhancement and tumor localization. To investigate the secondary thermal neutron production, MC simulations were developed to estimate the number of neutron capture reactions with Gd for a wide variety of charged particles including protons, helium, carbon, nitrogen, oxygen, neon, silicon, argon, antiprotons and negative pions. Within this study, all selected charged particles have been considered or proposed for clinical use in radiation therapy of cancers. We simulated two different SOBP depths and ranges for various particles on a soft tissue box containing a gadolinium contrast agent (GDCA) infused tumor located in the center of each SOBP peak. Since the physics used to produce secondary thermal neutron production is often susceptible to misrepresentations in particle transportation, a secondary objective was included to incorporate Geant4 as an independent verification of the results produced from MCNP6. The results from this study compare the amount of Gd neutron captures produced per absolute and RBE weighted dose for 10 different particles. Therefore, we can identify which particle would clinically benefit the most from Gd spectroscopic imaging and dose enhancement.

## Materials and methods

### MCNP particle simulations

We used two of the most widely used and well-benchmarked MC codes for heavy charged particle transport in this study: Monte Carlo N-Particle 6 (MCNP Version 6.1.1, by LANL) a general purpose radiation transport code and a second validation using GEometry ANd Tracking (Geant4 Vr. 10.02.p02) MC toolkit^32^.

We investigated two different SOBP configurations. In the first, we used a 5–10 cm SOBP with 5 × 5 cm^2^ field size (FS) incident on a 30 × 30 × 30 cm^3^ box of ICRU Soft Tissue having four components (H 10.1%, C 11.1%, N 2.6%, O 76.2% and a tissue density of 1.00 g/cm^3^) for all incident particles. Located in the center of the SOBP, an 8 cm^3^ volume of 3 mg/g (3,000 parts per million (ppm)) Gd solution was created and placed along the 6.5–8.5 cm depth of the soft tissue box to represent a Gd infused tumor.

To investigate the effect of GdNC with the increase of treatment depth, a second configuration with 10–15 cm SOBP, 5 × 5 cm^2^ FS, was employed for three particles (proton, helium, carbon). We investigated these three particles as they are the most clinically relevant particles, in addition, these particles can be compared to the simulation work published by Safavi-Naeini et al.

Using MCNP for these configurations, total dose, total neutron spectrum, thermal neutron production and the total Gd capture reaction rate were scored within the tumor. Secondary particle transport such as protons, neutrons, electrons, photons, heavy ions, alpha particles, tritons, helium ions and deuterons were considered in the simulation, except for antiprotons where both negative and positive muons, pions and kaons were also included.

Within MCNP, the dose was scored in two separate ways: using the + F6 energy deposition tally with all particle transport on except for photons, and using the F8* energy deposition tally measuring only the photon dose component. This is a requirement in order to obtain accurate results for photon dose as the F6 measurement tally assumes energy is deposited locally, which is not the case for secondary electrons released for photon interactions. For MCNP, the total neutron spectrum and thermal neutral fluence were determined using the F4 cell flux tally for neutrons. To determine the Gd neutron capture reaction rate, the FM tally multiplier was used. The total dose, thermal neutron fluence and Gd neutron capture rate were determined to within a statistical uncertainty of < 1% and the total neutron fluence to within 5%. The neutron spectrum for antiprotons, negative pions, protons, and helium ions, was scored in logarithmic bins ranging in neutron energy from 1 × 10^–8^ up to a maximum of 600 MeV. For heavier ions such as, carbon, nitrogen, neon, oxygen, silicon and argon, logarithmic bins were extended to 1.6 × 10^4^ MeV.

The physics used to produce secondary thermal neutron production from 10 different particles are complex and susceptible to particle transportation misrepresentations from selected models. To mitigate erroneous results, an additional MC toolkit, Geant4, was incorporated into this study to provide an independent check of the results from MCNP6. Geant4 simulation outputs were determined using the G4ScoringManager (UI) interface over the tumor volume for the thermal neutron fluence and total dose deposited. To determine the uncertainty value of each output for Geant4, five sets of simulations were performed by varying the random number seed. The standard deviation of the mean of each output was quantified and found to be (< 5%). All secondary particles were transported throughout the Geant4 simulations.

### Spread-out Bragg peak simulations

To approximate the SOBP produced from an active scanning beam, an analytical formalism was used to produce a proton SOBP from Jette et al.^33^. The formalism calculates the beam energies with respective weights for the depth and range of a proton beam SOBP. To determine the beam energy weights to build the SOBP of another particle, a relationship linking the heavy charged particle (HCP) continuous slowing down approximation range (${R}_{CSDA}^{Mi}$) to that of a proton (${R}_{CSDA}^{p}$) can be established as below^34^:

1 $$R_{CSDA}^{Mi} \left( {KE_{i} } \right) = \left[ {\left( {\frac{{Z^{2} }}{{z_{i}^{2} }}} \right)\left( {\frac{{M_{i} }}{{m_{p} }}} \right)} \right]\left[ {R_{CSDA}^{p} \left[ {\left( {\frac{{m_{p} }}{{M_{i} }}} \right) \times (KE_{i} )} \right]} \right]$$

where *Z* is the proton atomic number, ${z}_{i}$ is the HCP atomic number, ${(KE}_{i})$ and ${(KE}_{p})$ are the initial kinetic energies for the HCP and proton, ${M}_{i}$ and ${m}_{p}$ are the mass of the HCP and proton, respectively.

To simulate the SOBP using MCNP, a 5 × 5 cm^2^ planar source was made incident on a 30 × 30 × 30 cm^3^ ICRU soft tissue box. For the 5–10 cm SOBP, voxels were created using 0.25 cm increments along the plateau and 0.1 cm increments along the Bragg peak beam direction and 2 × 2 cm^2^ in the direction perpendicular to the ion beam. For the 10–15 cm SOBP, voxels of 0.5 cm increments were created within the plateau and 0.2 cm increments along the Bragg peak in the beam direction. The simulation was continued until the dose calculation uncertainty of < 1% was achieved inside the voxels. Within Geant4, the SOBP dose was scored in voxels (0.1 × 2 × 2 cm^3^) of 0.1 cm resolution along the beam path. The dose distributions for each setup and particle were qualitatively compared and found to follow a similar depth dose distribution between the two MC codes. Table 2 displays the maximum and minimum particle energies in MeV per nucleon (MeV/u) used to produce each SOBP.

**Table 2:** Energy ranges of incident particle beams to produce a SOBP in each case.

Particle	Particle (5 to 10 cm SOBP)		Particle (10 to 15 cm SOBP)	
	Min E (MeV/u)	Max E (MeV/u)	Min E (MeV/u)	Max E (MeV/u)
Proton	80	117	117	147
Helium	80	117	117	147
Carbon	147	216	217	276
Nitrogen	160	236	–	–
Oxygen	173	255	–	–
Neon	196	289	–	–
Silicon	237	350	–	–
Argon	249	399	–	–
Antiproton	80	117	–	–
Negative Pion	34	52	–	–

#### MCNP6 particle physics models

The MCNP6 continuous-energy neutron data libraries with the most recent ENDF material card were selected. For the simulations using a proton source, the LA150 NJOY proton data libraries were used when available, otherwise the default Cascade-Exciton Model (CEM) was used. For the particle simulations other than protons, the Los Alamos Quark Gluon String Model (LAQGSM) was employed to simulate the nuclear collisions produced from heavy charged particles. The LAQGSM model describes the stages of nuclear interactions and is valid over a high energy range up to 1 TeV^35^.

#### Geant4 particle physics models

For the Geant4 simulations, the full list of selected physics models are provided in Table 3. The primary hadronic physics list selected is QGSP_BIC_HP. QGSP uses the quark gluon string model (QGSP) for high energy interactions of protons, neutrons, pions, kaons and various nuclei. The high-energy interaction creates an excited nucleus, which is passed on to the pre-compound model, which models nuclear de-excitation. QGSP_BIC uses the G4 Binary cascade for primary protons and neutrons with energies below ~ 10 GeV. Binary cascade has been validated to describe the production of secondary particles produced in interactions of protons and neutrons with nuclei. QGSP_BIC also uses the binary light ion cascade to model the inelastic interactions of ions up to a few GeV/nucleon with matter^36^.

**Table 3:** Geant4 physics models for all particle simulations.

Interaction	Energy range	Geant4 model
Electromagnetic interaction	0–10 GeV	G4EmStandardPhysics_option3
Radioactive decay	N/A	G4RadioactiveDecayPhysics
Particle decay	N/A	G4Decay
Hadron elastic	0–100 TeV	G4HadronElasticPhysicsHP
Ion inelastic	0–110 MeV	Binary Light Ion Cascade
	100 MeV–10 GeV	QMDModel
Neutron capture	0–20 MeV	NeutronHPCapture
	19.9 MeV–100 TeV	nRadCapture
Neutron inelastic	0–20 MeV	NeutronHPInelastic
	19.9 MeV–9.9 GeV	Binary Cascade
Neutron elastic	0–4 eV	NeutronHPThermalScattering
	4 eV–20 MeV	NeutronHPElastic
	20 MeV–100 TeV	hElasticCHIPS
Proton inelastic	0–9.9 GeV	Binary Cascade

The list QGSP_BIC_HP has the addition of the high precision neutron package (NeutronHP) to transport neutrons below 20 MeV down to thermal energies^37^. The recommendation to use the high precision neutron packages for each neutron interaction process was followed as these lists originate from evaluated nuclear data files (ENDF), which have been extensively validated. Similar to MCNP, the thermal energy neutron elastic scattering files S(α,β), from ENDF/B-VII, were used for the thermal neutron scattering of H in the ICRU soft tissue phantom.

## Results

### Particle depth dose distributions

**Figure 1:**
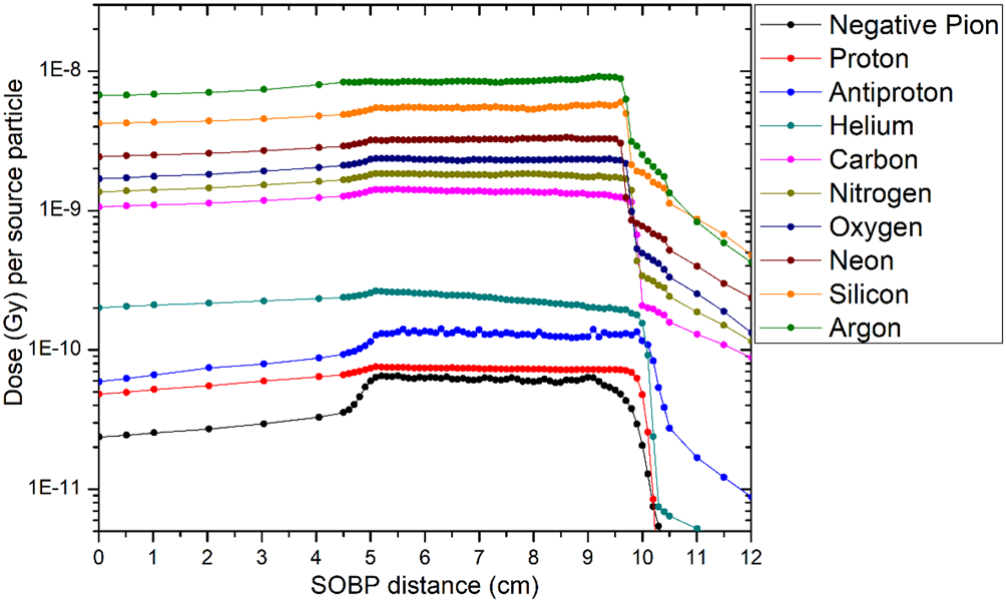
Simulated SOBP dose distributions for 5–10 cm SOBP using MCNP6.

The 5–10 cm SOBP distributions for all 10 investigated particles are shown in Fig. 1. The dose distributions were produced within MCNP6 and measured as a function of dose in Gy per source particle. It is evident from the figure that fragmentation contributes to dose beyond the SOBP for all heavy ions from carbon onward as previously reported^37^. Also, the expected dose halo^38^ from antiprotons is evident from the long tail of the SOBP.

**Figure 2:**
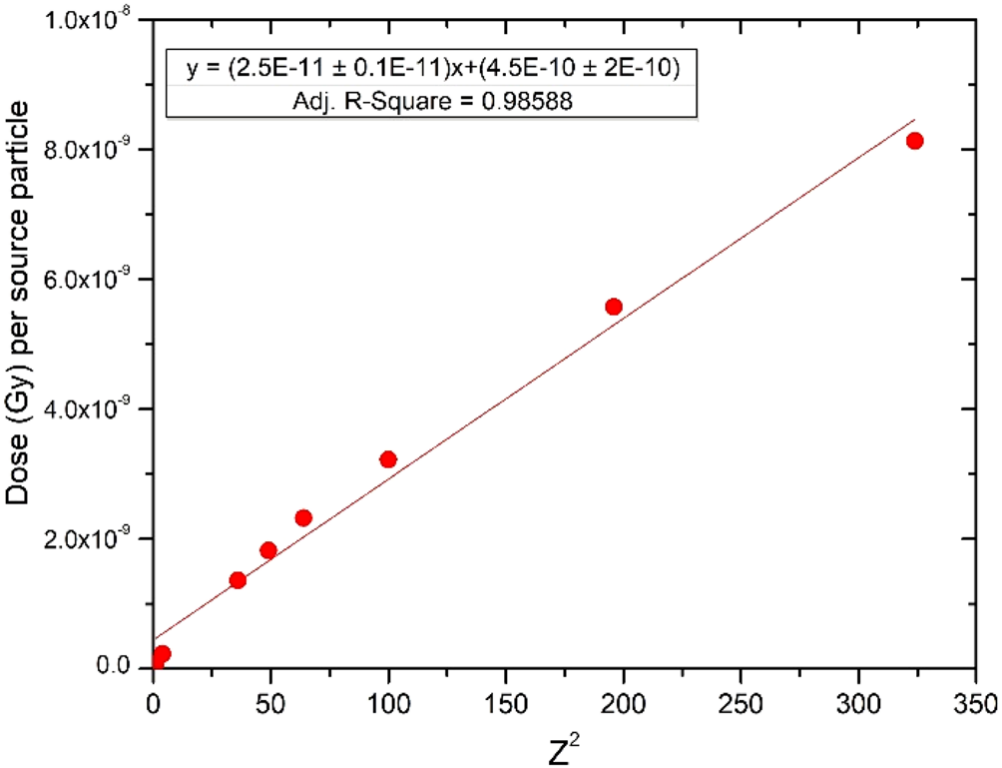
Dose per source particle versus atomic number squared (Z^2^). The relationship is linear as expected based on the Z^2^ dependence of the Bethe stopping power equation. A statistically significant slope was determined (p < 0.001).

Argon (Ar) produced the largest amount of dose per source particle due the large atomic number (Z = 18). This is expected based on the Z^2^ dependence of the Bethe stopping power equation. The dose per source particle is found to increase linearly with Z^2^ for all ions except antiprotons and negative pions. A plot displaying dose as a function of Z^2^ is shown in Fig. 2. Antiprotons and negative pions were excluded from the plot as these particles produce more complex dose distributions due to the uniqueness of their interactions.

**Figure 3:**
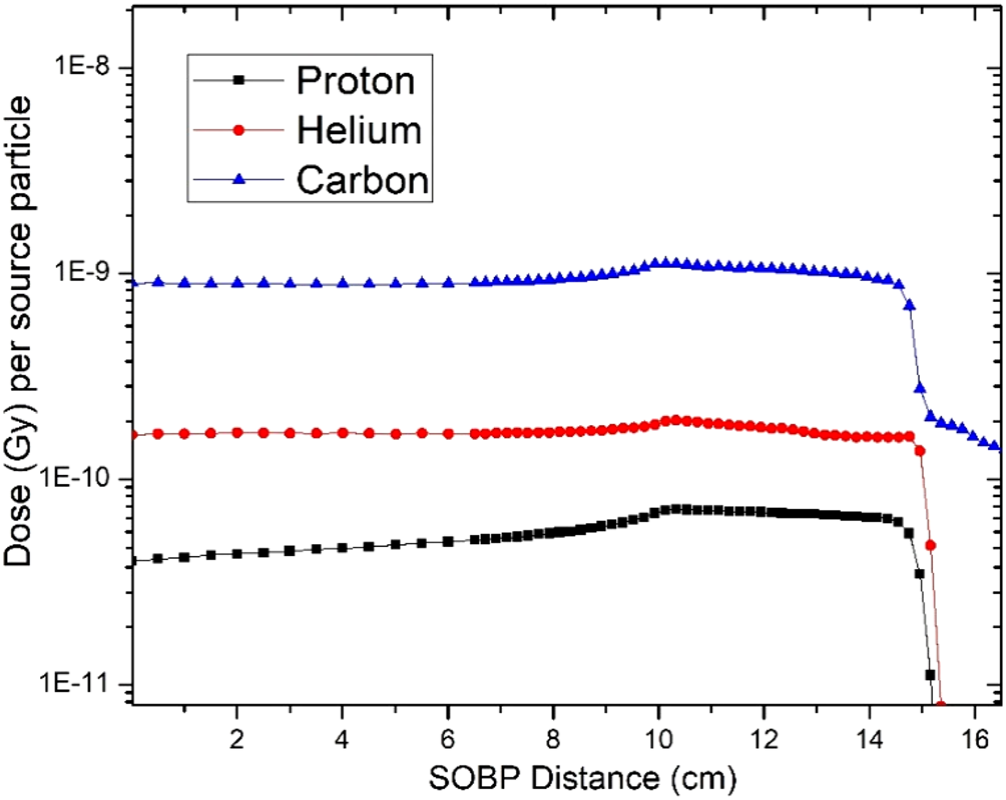
Simulated dose distributions for 10–15 cm spread-out Bragg peaks from MCNP6 for protons, helium and carbon ions.

The smallest dose per source particle was produced from the lightest ion investigated, negative pions (− π), which is slightly less than protons over the Bragg peak region. The peak-to-plateau dose (Gy) for each particle can be seen within Table 4. Antiprotons produced a much larger dose over the Bragg peak compared to protons. At the center of the SOBP (7.5 cm depth), the physical dose from antiprotons is 1.8 times higher than protons. A study validating experimental antiproton measurements with MC toolkits (FLUKA, SHIELD-HIT) conducted by Bassler et al. found that antiprotons have roughly 2 times higher physical dose than protons^39^. Our results are in agreement with the reported findings. Studies have also been conducted comparing the antiproton dose characteristics between FLUKA and MCNP and have shown close agreement^40^. The SOBP distributions from 10 to 15 cm for protons, helium ions, and carbon ions are shown in Fig. 3.

**Table 4:** Peak-to-plateau dose for each particle and geometry.

Particle (5 to 10 cm SOBP)	Argon	Silicon	Neon	Oxygen	Nitrogen
Peak-to-plateau dose	1.25	1.33	1.33	1.35	1.29
Particle (5 to 10 cm SOBP)	Carbon	Helium	Proton	Antiproton	Negative Pion
Peak-to-plateau dose	1.27	1.15	1.52	2.20	2.58
Particle (10 to 15 cm SOBP)	Carbon	Helium	Proton	–	–
Peak-to-plateau dose	1.16	1.08	1.71	–	–

### Neutron production

The thermal neutron fluence, total neutron fluence, total amount of Gd neutron captures and the Gd neutron capture rate normalized to protons are shown in Table 5. For simplicity, all values have been normalized to the absolute dose in Gy.

**Table 5:** Simulated neutron fluence and Gd neutron capture reactions per Gy of absolute dose for the various charged particles investigated in this study. The last column is the Gd neutron capture rate relative to protons.

Particle 5 to 10 cm (SOBP)	Thermal neutron fluence (cm^-2^/Gy)	Total neutron fluence (cm^-2^ /Gy)	Gd neutron capture reactions (captures/Gy)	Normalized Gd neutron capture reactions (captures/Gy)
Argon	1.7 × 10^5^	1.2 × 10^7^	5.0 × 10^5^	0.24
Silicon	1.7 × 10^5^	9.5 × 10^6^	5.0 × 10^5^	0.24
Neon	2.2 × 10^5^	1.1 × 10^7^	6.5 × 10^5^	0.30
Oxygen	2.6 × 10^5^	1.2 × 10^7^	7.4 × 10^5^	0.34
Nitrogen	3.1 × 10^5^	1.4 × 10^7^	8.9 × 10^5^	0.42
Carbon	3.7 × 10^5^	1.5 × 10^7^	1.1 × 10^6^	0.49
Helium	1.3 × 10^6^	3.3 × 10^7^	3.7 × 10^6^	1.7
Proton	7.4 × 10^5^	1.1 × 10^7^	2.1 × 10^6^	1.0
Antiproton	1.4 × 10^7^	2.8 × 10^8^	3.7 × 10^7^	18
Negative Pion	2.9 × 10^7^	6.9 × 10^8^	8.3 × 10^7^	39
**10 to 15 cm (SOBP)**				
Carbon	7.1 × 10^5^	2.4 × 10^7^	4.5 × 10^6^	0.56
Helium	2.1 × 10^6^	4.7 × 10^7^	1.1 × 10^7^	1.7
Proton	1.3 × 10^6^	1.4 × 10^7^	5.8 × 10^6^	1.0

For the 5–10 cm SOBP, antiprotons and negative pions were associated with the largest neutron production and largest number of Gd neutron captures. The third largest was helium particles. For the 10–15 cm SOBP, helium particles produced the largest number of Gd neutron captures. Table 6 shows Gd neutron capture results normalized to RBE weighted dose in Gray Equivalent (GyE). It is widely known that the RBE in charged particle therapy is one of the greatest sources of uncertainty^41^ and can vary based on many factors including location within the Bragg peak^42^. However, we chose representative values from the literature for these comparisons. The last column in Table 6 consists of the ratio of neutron capture per GyE normalized to protons.

**Table 6:** Simulated Gd neutron capture reactions normalized to an estimate of RBE weighted dose (RBE × Gy). The last column is RBE weighted captures normalized to protons.

RBE	RBE value reference	Particle 5 to 10 cm (SOBP)	Gd neutron captures reactions per RBE × Gy (captures/GyE)	Normalized RBE weighted Gd neutron capture reactions
4.25	Goldstein et al.^42^	Argon	1.2 × 10^5^	0.06
3.75	Blakely et al.^44^	Silicon	1.3 × 10^5^	0.07
3.5	Goldstein et al.^42^	Neon	1.9 × 10^5^	0.10
3.25	Tran et al.^45^	Oxygen	2.3 × 10^5^	0.12
3	Tran et al.^45^	Nitrogen	3.0 × 10^5^	0.15
2.75	Goldstein et al.^42^	Carbon	3.8 × 10^5^	0.20
1.5	Goldstein et al.^42^	Helium	2.5 × 10^6^	1.3
1.1	Tepper et al.^43^	Proton	1.9 × 10^6^	1.0
2	Holzscheiter et al.^46^	Antiproton	1.9 × 10^7^	10
2.5	Raju et al.^31^	Negative Pion	3.3 × 10^7^	17
**10 to 15 cm (SOBP)**				
2.75	Goldstein et al.^42^	Carbon	1.6 × 10^6^	0.2
1.5	Goldstein et al.^42^	Helium	7.2 × 10^6^	1.2
1.1	Tepper et al.^43^	Proton	5.3 × 10^6^	1.0

**Figure 4:**
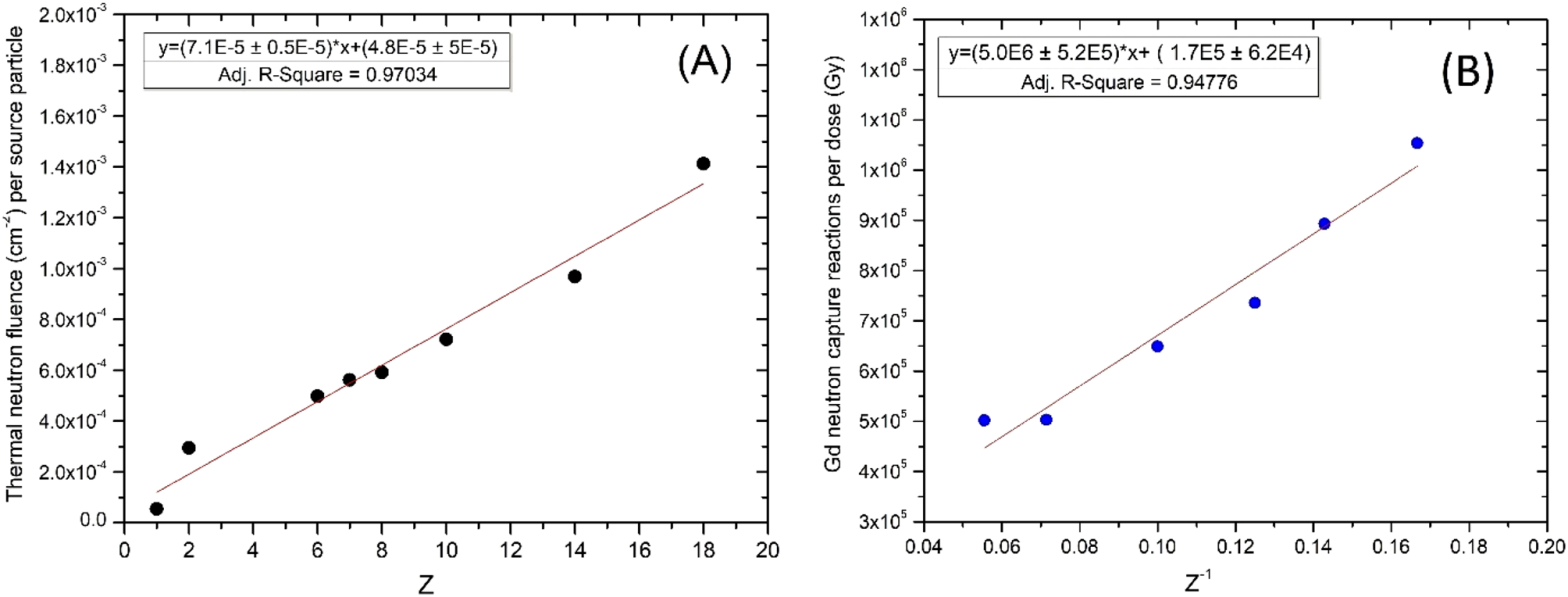
(**A**) Thermal neutron production per source particle as a function of atomic number (Z), (**B**) Gadolinium neutron capture reactions per Gy as a function of 1/atomic number (Z).

The thermal neutron fluence was found to scale linearly with the atomic number for all particles except antiprotons, and negative pions. This is shown in Fig. 4A. Since the dose is proportional to Z^2^, and the thermal neutron fluence is proportional to Z, the ratio of thermal neutron captures to dose was found to be proportional to 1/Z for heavy charged particles as shown by the linear relationship in Fig. 4B. Protons, helium ions, antiprotons and negative pions were excluded from Fig. 4B due to their lower Z number. The r^2^ values from both fits in Fig. 4 demonstrate strong linear correlations with a positive slope (p < 0.001).

The comparison of thermal neutron fluence per absorbed dose (Gy) between both MC codes, MCNP and Geant4, is provided in Table 7. All simulations were found to be within one order of magnitude. The agreement is reasonable regardless of the subtleties of the physics models and computational implementation between the two systems. The highest thermal neutron production originating from both antiprotons and negative pions is evident in both codes. Similarly, the trend of increased thermal neutron fluence per Gy with decreasing atomic number can be observed for the two codes.

**Table 7:** Comparison of MCNP and Geant4 MC results.

Particle 5 to 10 cm (SOBP)	MCNP6 Vr. 6.1.1	Geant4 Vr. 10.02.p02	Ratio
	Thermal neutron fluence (cm^−2^/Gy)	Thermal neutron fluence (cm^−2^/Gy)	
Argon	1.74 × 10^5^	2.81 × 10^5^	1.62
Silicon	1.74 × 10^5^	2.01 × 10^5^	1.15
Neon	2.24 × 10^5^	2.52 × 10^5^	1.13
Oxygen	2.55 × 10^5^	3.99 × 10^5^	1.57
Nitrogen	3.09 × 10^5^	3.75 × 10^5^	1.22
Carbon	3.66 × 10^5^	3.95 × 10^5^	1.08
Helium	1.27 × 10^6^	1.73 × 10^6^	1.36
Proton	7.40 × 10^5^	8.64 × 10^5^	1.17
Antiproton	1.36 × 10^7^	7.26 × 10^6^	0.56
Negative Pion	2.89 × 10^7^	4.28 × 10^6^	0.14
**10 to 15 cm SOBP**			
Carbon	7.05 × 10^5^	9.66 × 10^5^	1.37
Helium	2.13 × 10^6^	2.19 × 10^6^	1.03
Proton	1.26 × 10^6^	1.49 × 10^6^	1.18

**Figure 5:**
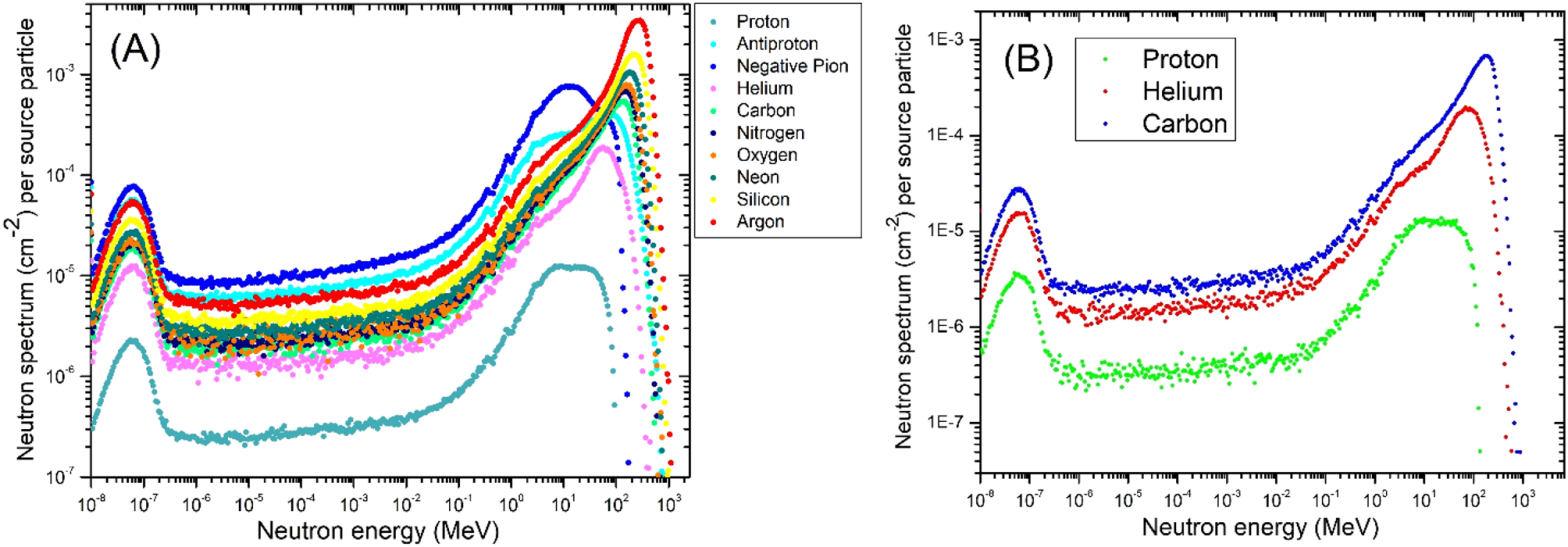
(**A**) Total neutron fluence spectrum for a 5 to 10 cm SOBP for all 10 particles. (**B**) Total neutron fluence spectrum for a 10 to 15 cm SOBP for protons, helium ions, and carbon ions.

The total neutron fluence spectra per source particle for each SOBP investigated can be observed in Fig. 5. Qualitatively, the neutron spectra has identical features consisting of a broad peak in the low energy (< 0.1 eV) region and at the high energy region. The profound neutron peak in the thermal energy region is important for the application of neutron capture techniques for each of the charged particles.

## Discussion

In this work, we investigated the Gd neutron capture rate for ten ion beams. Negative pions, antiprotons, and helium ions produced a Gd neutron capture per GyE value larger than that of the protons. Although, studies have been conducted investigating the feasibility of using both antiprotons and negative pions for therapy, helium ions may be the only clinically viable particle out of the three^37,38,47,48^. In a study conducted by Paganetti et al., it was determined that although 1 × 1 cm^2^ antiproton beam characteristics are comparable to that of protons, a clinically relevant field size of 10 × 10 cm^2^ would be inferior^38^. This is because the majority of the annihilation energy from antiprotons is given to the long-range secondary particles, which result in a dose halo around the primary field and degrade both the lateral and distal fall-off^38^. Negative pions have been studied in the context of several different clinical trials. The investigations concluded that though the negative pions lead to an effective treatment, they add little to no benefit when compared to other ion therapies^47^. Three separate institutes, Los Alamos Meson Physics Facility (LAMPF), Tri University Meson Facility (TRIUMF) and the Swiss Institute for Nuclear Research (SIN-PSI) have performed a total of 245 negative pion treatments for prostate cancer. Results varied throughout each institution, the most recent study conducted at SIN-PSI demonstrated a local control of 89% after 2 years and 83% after 5 years. A major concern with negative pion therapy is the justification for the large therapeutic treatment cost^47^. While there are arguably other secondary particles that can be used for imaging in antiproton^49^ and negative pion therapy^50^, these particles are correlated to deposited dose, whereas imaging Gd would identify characteristics of the tumor volume. This dynamic tumor image displayed in coincidence to treatment can be fused with a CT or MRI image series and used as a method to localize tumor position. The visible tumor image can be used as a method to indicate the effectiveness of treatment with each subsequent fractionation and provide spatial information for adaptive therapy techniques^9^. Also, dose enhancement was not investigated within this study, however, it is worth further investigation to determine if Gd dose enhancement would provide an impactful benefit to therapeutic treatment from any of these 10 particles.

Recently, there has been a strong interest in the application of helium ions for treatment of cancer^48^. Helium exhibits depth dose characteristics identical to protons along the longitudinal depth, but has reduced lateral scattering due to its larger mass. This advantage is interesting as the LET of helium ions is higher than that of protons^45^. Distinct from carbon and other heavy ions, helium produces little to no fragmentation tail and therefore treatments are capable of gaining the benefit of a higher RBE value while also maintaining a sharp dose fall-off after the tumor^48^.

It was determined in this study that heavier ions were less than ideal for thermal neutron capture techniques, particularly, when the neutron production was scaled in terms of relative biological dose. This result, although not advantageous for neutron capture based imaging, is beneficial due to a reduced neutron dose to healthy structures for heavier ions.

To model the various ions used within this study, two Monte Carlo codes were selected and found to be within one order of magnitude for each particle and measurement setup. This result comparing the Geant4 toolkit with the MCNP6 transport code for the production of thermal neutron fluence over absolute dose relies heavily on the physics models implemented in each simulation. Regardless, this study determined a consistently close agreement in relative magnitude for the measurement of thermal neutron fluence per absorbed dose (cm^−2^/Gy) for each of the simulated particles.

In our previous feasibility study (Van Delinder et al*.* 2020), we studied for the first-time, experimental measurements of the Gd neutron capture spectrum on a proton therapy treatment unit. Five measurements were obtained in which a signal-to-noise ratio of greater than 5 was determined to be achievable for imaging Gd in proton therapy for high dose per fraction treatments^9^. From our simulation results, it can be concluded that these values would be larger for a passive treatment technique that utilizes helium particles, negative pions or antiprotons.

A recent study by Safavi-Naeini et al. investigated tumor dose enhancement as a result of thermal neutron capture in Gd for ion therapy. A 10–15 cm proton SOBP administered on a box of PMMA with a 5 cm^3^ tumor resulted in an average fluence of roughly 8 × 10^8^ thermal neutrons per Gy (cm^−2^/Gy) of dose via simulation^7^. This thermal neutron production is significantly higher than the results obtained in our work using ICRU soft-tissue, as well as our previous study using a water phantom^9^. Based on this, there is a need for a Monte Carlo study investigating neutron production with variation of SOBP by field size and Gaussian pencil beam on several different tissue-like materials. Since neutron production is directly proportional to the specifics of the treatment unit, beam configuration, tissue heterogeneity and other components; a more comprehensive study would incorporate a scaling or validation from experimental measurements. Regardless, the increased thermal neutron production, which was determined from Safavi-Naeini et al. would result in a greater amount of Gd neutron captures and therefore, provide a greater benefit for the spectroscopic application of PNGXD as well as Gd neutron capture dose enhancement. A large thermal neutron production would have dual benefits: allowing an increased localized tumor dose and the capability to image the enhanced dose component from the PNGXD photons.

## Conclusions

Ten different particles were simulated to study thermal neutron production for the application of Gd neutron capture imaging (PNGXD) and Gd neutron capture therapy. Excluding three particles (antiprotons, negative pions, helium ions), a trend was observed demonstrating a decrease in neutron production per GyE with increasing atomic number. We found that three particles, antiprotons, negative pions, and helium ions, consistently produced more thermal neutron captures than protons. For a 5 to 10 cm SOBP, these three particles produced 1.9 × 10^7^, 3.3 × 10^7^, 2.5 × 10^6^ neutron captures per GyE, respectively. When normalized to protons per RBE weighted dose, Gd neutron capture ratios of 10, 17 and 1.3 were determined in antiprotons, negative pions, and helium ions. It is likely that these three particles would benefit the most from PNGXD imaging and tumor dose enhancement, however, helium ions continue to be the most clinically attractive particle out of the three.
